# Spatiotemporal patterns of spontaneous brain activity: a mini-review

**DOI:** 10.1117/1.NPh.9.3.032209

**Published:** 2022-04-12

**Authors:** Lisa Meyer-Baese, Harrison Watters, Shella Keilholz

**Affiliations:** a Emory University, Georgia Institute of Technology, Wallace H. Coulter Department of Biomedical Engineering, Atlanta, Georgia, United States; b Emory University, Neuroscience, Atlanta, Georgia, United States

**Keywords:** electrophysiology, magnetic resonance imaging, neuroimaging, optical imaging, spatiotemporal patterns

## Abstract

The brain exists in a state of constant activity in the absence of any external sensory input. The spatiotemporal patterns of this spontaneous brain activity have been studied using various recording and imaging techniques. This has enabled considerable progress to be made in elucidating the cellular and network mechanisms that are involved in the observed spatiotemporal dynamics. This mini-review outlines different spatiotemporal dynamic patterns that have been identified in four commonly used modalities: electrophysiological recordings, optical imaging, functional magnetic resonance imaging, and electroencephalography. Signal sources for each modality, possible sources of the observed dynamics, and future directions are also discussed.

## 1 Introduction

From molecules to the whole brain, constant fluctuations in brain activity occur at every scale of organization. The modeling of these constantly changing activity patterns in the brain, or spatiotemporal patterns, has become a major focus in recent years. The relative timing of these fluctuations within a neural population is known to be constantly changing. Instead of having precise synchrony, a range of phase offsets is possible. With flexible phase offsets, these fluctuations in neural activity can be viewed as traveling waves of different shapes including plane, radial, and spiral waves.^1^ The combination of multiple traveling waves forms subsequent complex spatiotemporal patterns.^1^ Moreover, these spatiotemporal patterns can recur repeatedly over time, sometimes rhythmically, sometimes at variable intervals. These waves travel along brain networks at multiple scales, modulating neural excitability as they pass. Troves of data have now been collected measuring the spatiotemporal dynamics of these traveling waves in different animal models and at different scales, but assessing the causality between patterns measured at different scales remains a major challenge. Relating these spatiotemporal dynamics across scales and modalities is critical for a better understanding of causality between microscopic and macroscopic brain activity and the etiology of behavior and disease.^2^^,^^3^

Relating spatiotemporal dynamics within scales is always simpler than relating across scales of organization.^4^ Activity across microscopic and macroscopic scales is related by what has been termed circular causality.^5^ This means that emergent patterns from smaller scales (i.e., single neurons or circuits) generate large scale patterns at higher levels of organization (systems or the whole brain), which in turn constrain the activity generated at smaller scales. A better understanding of this circular causality across brain hierarchy, in both health and pathology, requires an integrated understanding of the spatiotemporal patterns across experimental modalities. As a first step toward the goal of cross-scale comparison, this review describes and compares patterns that occur at different spatial and temporal scales, measured with different modalities.

A wide range of characteristic spatiotemporal dynamic patterns has been identified at different scales, from single neurons to systems to whole brains. In this review, we discuss systems-level spatiotemporal patterns (primarily cortex-wide scale patterns) in different modalities. Specifically, we survey the main spatiotemporal patterns that have been detected at the mesoscale using electrode arrays and optical imaging and at the macroscale with functional magnetic resonance imaging (fMRI) and electroencephalography (EEG). For a brief overview and comparison of these modalities see Table 1.

**Table 1:** Overview of the spatiotemporal resolution, specificity, observed patterns, and considerations for all of the discussed imaging modalities.

	Electrode arrays	Optical imaging of fluorescence and *intrinsic signals*	fMRI	EEG
Number of neurons	One to hundreds	Tens to hundreds	n/a	Millions, cortical surface
		*n/a*		
Spatial resolution	200 to 400 μm	50 to 100 μm	400 to 500 μm	10 to 20 mm
		∼100 μm		
Time resolution	Millisecond	Millisecond (depends on kinetics of sensor)	Second	Millisecond to hundreds of milliseconds
		*Second*		
Cell-type specificity	Limited	High (depends on the promoter)	Very low signal from hemodynamic response to neural activity.	Low signal predominantly from EPSPs and IPSPs in cortical pyramidal apical dendrites
		*n/a*		
Patterns found	(1) Slow oscillation generated around layer 5 (*in vitro* ferret occipital cortex, *in vivo* rats and cats) ^6^^,^^7^^,^^8^^,^^9^^,^^10^	(1) Global plane waves traveling anterior to posterior (mice)^13^^,^^14^	(1) Propagating activity, such as QPPs (rats and humans)^16^^,^^17^^,^^18^^–^^22^	(1) Transient spatial configurations, such as EEG and microstates (humans)^31^^,^^26^^,^^27^^,^^28^^,^^29^^,^^30^
	(2) Brief spontaneous depolarizations, localized to an area of a barrel column (rats and mice)^11^	(2) Standing waves that have no net movement (mice)^15^	(2) Transient spatial configurations, such as CAPs (rats and humans)^23^^,^^16^^,^^24^^,^^25^	
	(3) Propagating waves that traveled horizontally across dorsal cortex (rats and mice)^11^^,^^12^	(3) Local source, sink, and saddle points (mice)^15^		
Pitfalls	(1) Large artifacts	(1) Problems of light delivery	(1) Low temporal resolution	(1) Low spatial resolution
	(2) Tissue damage	(2) Dependent on efficiency of viral transfection	(2) No cell type specificity	(2) Signal primarily from postsynaptic potentials of apical dendrites
		(3) Potential toxicity of opsins	(3) Low SNR	(3) Superficial signal, no localization at greater cortical depths
		(4) Unspecific effects		

A better understanding of how systems-level dynamic patterns vary across modalities and scales will enhance knowledge of the brain’s functional architecture, giving insight into the origins of intentional behavior and the neural correlates of consciousness, and ultimately allowing for a better understanding of the etiology of pathological brain states and disease.

## 2 Electrode Arrays

### 2.1 Source of Signal

Single electrode recording has been in use for decades, but recent advances to multi-electrode arrays have enabled researchers to characterize the macroscopic dynamic patterns. Electrode arrays can be used as a tool to study spatiotemporal dynamics by measuring changes in local field potentials (LFPs). LFPs have several qualities that make them suitable for studying neural brain dynamics. LFPs represent the aggregate activity of small populations of neurons characterized by their extracellular potentials.^32^ A cardinal feature of this signal is that it represents activity from many neurons in the vicinity of the recording site. Thus, it samples local neuronal populations and captures network dynamics that would otherwise be missed by single-cell recordings.^33^ These network dynamics are what define LFP, which has a spatial resolution of 200 to 400  μm and a temporal resolution on the order of millisecond. A drawback to LFPs is that the spatial coverage is limited to a single column or activity across distinct columns with multiple arrays. Because of this, most of the identified dynamic patterns inform the localized occurrence and spread of spontaneous activity rather than a brain-wide understanding of spatiotemporal dynamics.

### 2.2 Dynamic Patterns

Electrode arrays have been used to identify recurring traveling waves in spontaneous activity in both cortical slices and surface recordings.^6^^,^^8^^–^^35^

*In vitro* experiments have proven to be beneficial for elucidating the mechanism that drives the observed recurrent propagation. Slow rhythmic activity (<1  Hz) was generated *in vitro* when slices of the ferret neocortex were placed in a bathing medium that mimics the extracellular ionic composition *in situ*.^6^ The transition from a depolarized to hyperpolarized state recurred with a periodicity of once every 3.44±1.76  s.^6^ These oscillations were initiated by layer 5 excitatory interactions between pyramidal neurons. For *in vitro* slice preparation, the slow oscillation then propagates within these infragranular layers.^6^ These oscillations were further studied across cortical slices using multi-electrode arrays to investigate local and global network dynamics. It was found that activation waves systematically propagated parallel to the cortical surface, with wide variability in spatial source and speed.^6^^,^^8^ Irrespective of their variability, wavefronts revealed loci across L4/L5, adding to the evidence that most excitable cell assemblies reside in L5.^6^^,^^7^^,^^8^

**Fig. 1:**
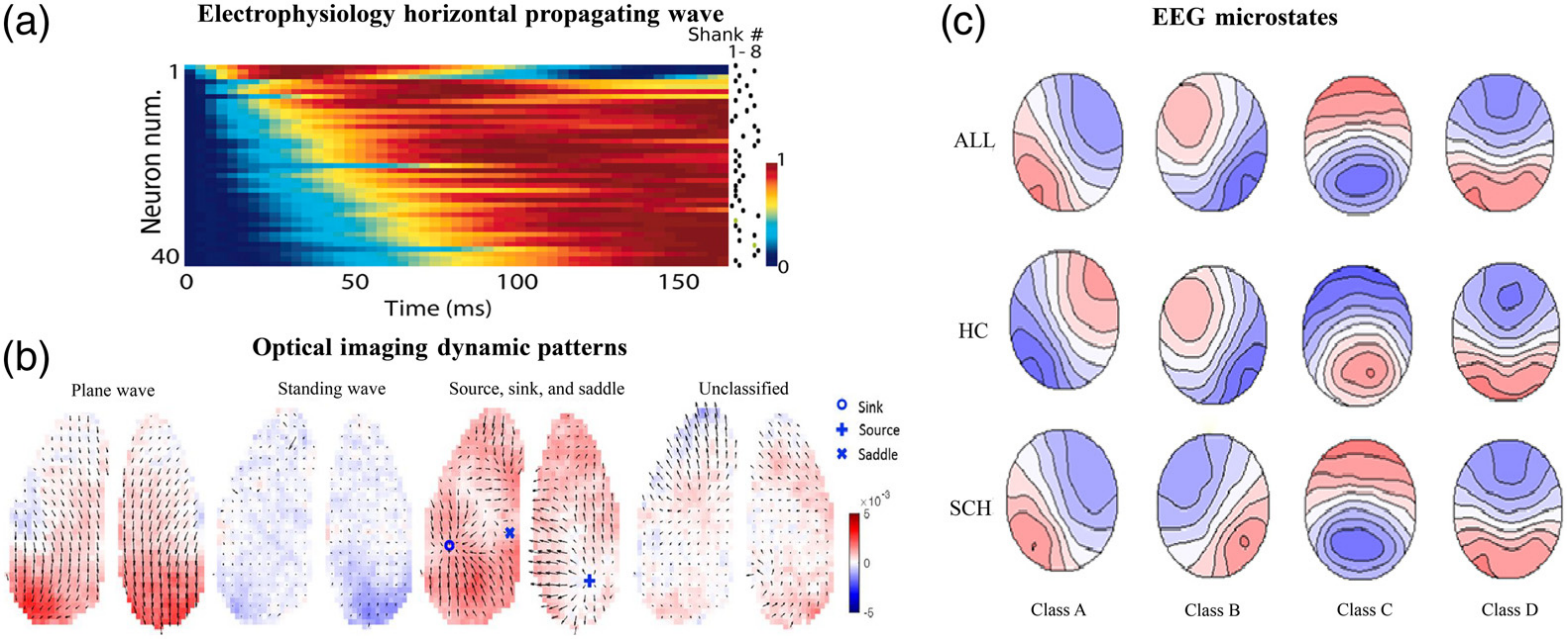
(a) Pseudocolor plot showing the normalized activity of a simultaneously recorded population triggered by “up” state onset, arranged vertically by latency. The dots indicate from which shank the neurons were recorded. Reprinted from Ref. 12 Copyright 2007 National Academy of Sciences. (b) Classification and detection of specific wave patterns at the cortex-wide scale. Reprinted from Ref. 15. (c) Spatial configuration of four microstate classes. SCH, individual with schizophrenia; HC, healthy controls; All, all participants in the study. Note how topographies extend over wide-scale areas representing global brain electrical events. Reprinted from Ref. 36.

Further evidence of the initiation souce in L5 was also found *in vivo* validating the role of layer ⅘ in “up” state initiation.^9^^,^^10^ Ensemble spatiotemporal dynamics of spontaneous depolarizations were either brief and localized to an area of a barrel column or occurred as propagating waves dependent on local glutamatergic synaptic transmission in layer ⅔.^11^ This flow of excitation is strictly feed forward from layer 4 neurons projecting to layer ⅔. Individual layer ⅔ pyramidal neurons are oriented preferentially along the rows of the barrel field, inducing preferential spread of excitation along the cortical axis.^11^ Neurons in the rat somatosensory cortex revealed that “up” state onsets initiate a sequential spread of activity across the population as shown in Fig. 1(a).^12^ They found that sequential structure was expressed only in the first ∼100  ms after the “up” state initiation and was most readily detected by the number of neurons with the highest firing rates (>2  Hz).^12^ A subset of “up” states then propagated as traveling waves. Regardless of their propagation direction, these traveling waves initiated similar local sequences. These patterns were found in both anesthetized and unanesthetized conditions.^12^ An observed difference between the two states was that the induced sequences under anesthesia lasted almost twice as long as those in unanesthetized animals.

Both *in vitro* and *in vivo* studies using electrode arrays have definitively demonstrated rhythmic activity that propagates along the cortex. These direct measurements of relatively localized neural activity help to validate the widespread patterns of propagation observed with other modalities and allow the mechanisms underlying the generation and coordination of such activity to be rigorously probed on a larger scale.

## 3 Optical Imaging

### 3.1 Source of Signal

An important component to studying the spatiotemporal dynamics of the brain is the understanding of the cellular mechanisms, something that cannot be done with LFPs. This includes the monitoring of membrane voltage at the cellular, circuit, and systems levels. A game-changing advance made in recent years using genetically engineered animal models has enabled the monitoring of these cellular mechanisms. These optical tools include voltage-sensitive dies and genetically encoded indicators of voltage (GEVIs) or calcium (GECIs). The set of optical imaging tools is cell type-specific, less invasive, and able to relate activity and anatomy and to facilitate long-term recordings of individual cell’s activities over weeks.^37^ Due to their inherent properties, there are key conceptual differences between GEVI and GECI signals. Things that must be considered are (1) the mode (calcium or voltage), (2) site of protein expression, and (3) sensitivity to action potential versus synaptic potential. GEVI signals largely represent the synaptic input arriving into a neural circuit whereas GECI signals present the action potential output generated in the neural circuit.^38^

Their differing sources, in turn, impact characteristics of the measured signal including the resolution and signal strength. The typical spatial resolution for population activity is 50 to 100  μm.^38^ The temporal resolution of calcium imaging is limited by calcium ion dynamics in the neurons and is usually too slow for following brain oscillations at frequencies >10  Hz, a limitation that is not shared by voltage imaging. Calcium indicators, however, usually produce larger fractional changes in fluorescence, whereas voltage-sensitive dyes provide smaller signals. For population imaging, with no cellular resolution, GECI optical signals showed an 8 to 20 times better signal-to-noise ratio (SNR) than GEVI signals.^38^

These tools can be combined with intrinsic optical signal imaging (IOSI), which makes use of the spectral properties of hemoglobin (Hgb), which has different absorption properties when oxygenated or deoxygenated, to measure changes in brain activity.^39^ IOSI benefits particularly from a relatively simplistic experimental setup in which there is no need for a genetically engineered animal. IOSI can provide information about hemodynamics that is complementary to the information about neural activity obtained with fluorescence imaging techniques.^40^

### 3.2 Dynamic Patterns

Optical imaging can measure activity from single neurons to cortex-wide spatiotemporal dynamics. Imaging cortex-wide patterns reveal critical neuronal dynamics that have unique spatiotemporal dynamics for slower and faster frequency bands of activity.^15^^,^^11^^,^^26^^,^^13^

Spontaneous infraslow activity (<0.1  Hz) travels through the cerebral cortex along stereotypical spatiotemporal trajectories that are recapitulated in Hgb optical imaging. The spatiotemporal trajectories of the infraslow calcium signal and the Hgb signals prove to be highly similar, demonstrating that neurovascular coupling is preserved during spontaneous activity.^13^ The time scale for this activity ranges from ±0.5  s. Under anesthesia, infraslow calcium signals travel posterior–anterior with posterior leading anterior activity by ∼0.5  s.^13^ This is consistent with other reports of correspondence between infraslow neural activity and hemodynamic signals.^41^^,^^42^ This relationship does not hold for slightly higher frequency bands of 1 to 4 Hz (Delta Band).^13^

Delta band spontaneous activity ranges from ±10  ms and travels in a reciprocal direction along the anterior–posterior axis of the mouse cortex. Under anesthesia, the anterior delta band leads the posterior delta by ∼100  ms.^13^ This result is consistent with others that have shown that delta activity travels anterior–posterior in anesthesia and sleep.^43^^,^^14^ Dynamics of the delta band were also investigated for anesthetized mice using voltage imaging.^15^ By treating the spontaneous neural activity as propagating waves, delta band activity is shown to propagate in heterogeneous directions. Specific wave patterns found included plane waves, standing waves, and source, sink, and saddle patterns [Fig. 1(b)]. Plane waves are those that travel anterior to posterior, whereas standing waves are defined as periods in which there is no apparent wave propagation across the analysis window.^15^ These patterns were observed globally and locally in mice, with local wave patterns of source, sink, or saddle emerging in preferred spatial locations.^15^

Most of the dynamic analysis that has been done so far using optical imaging has been confined to two frequency bands: infraslow activity and the delta band. It was found that infraslow motifs (<0.1  Hz) are analogous to cortical motifs observed from slow frequency spontaneous activity (0.5 to 6.0 Hz),^22^ in contrast to prior studies that showed the direction of propagation was different for delta and infraslow waves. A clear area for future research is determining how the spatiotemporal dynamics of these two bands interact. This is particularly important given that these slow frequencies are relevant for interpreting fMRI.

## 4 Functional Magnetic Resonance Imaging

### 4.1 Source of Signal

Due to its wide availability, intrinsic contrast mechanism, and ability to capture whole brain changes in activation, fMRI is one of the most widely used functional imaging techniques in humans and other animals.^44^

Most fMRI studies rely on blood-oxygen-level-dependent, or BOLD contrast, as an indirect measure of neural activity in the brain.^45^ The BOLD signal is a composite hemodynamic signal that is affected by factors including cerebral blood flow (CBF) and changes in the ratio of diamagnetic oxyhemoglobin to paramagnetic deoxyhemoglobin (HbR) in response to the increased metabolic demand of neurons and other cells in the brain. The increase of HbR in the wake of neural activity can be felt by the local protons in water, affecting their T2 and T2* relaxation times, resulting in an MRI-based signal that can be used for functional imaging.^44^^–^^47^ In other words, the BOLD signal that is almost universally used in fMRI studies is an indirect measure of neural activity.

The BOLD signal directly constrains the temporal and spatial resolution of fMRI and has important implications for the use of fMRI to study the spatiotemporal dynamics of the brain. The BOLD signal has a temporal resolution on the scale of the hemodynamic response to neural activation, which occurs on the order of a few seconds after neural activity. In humans, the hemodynamic response typically has a width of about 3 s and peaks between 5 and 6 s, but this is variable across conditions and animal models.^44^ The spatial resolution of BOLD fMRI (and the SNR) is dependent on the strength of the applied magnetic field.^44^^,^^48^ The typical voxel size in fMRI can range from 3 to 4 mm in lower field strength (1.5 to 3 T) magnets and from 400 to 500  μm with field strengths of 7 T and above.^44^^,^^48^

### 4.2 Dynamic fMRI Patterns

#### 4.2.1 Quasi-periodic patterns

Quasi-periodic patterns, or QPPs, are reliably recurring spatiotemporal patterns of propagating activity.^16^^,^^17^^,^^18^ The patterns are detected in fMRI data [typically resting state (rs)] using a correlation-based iterative approach without a user-defined start point (for more details, see Ref. 17). QPPs are an infraslow pattern (below 0.5 Hz), occurring on a timescale of 0.2 to 0.1 Hz, or 1 cycle every 5 to 10 s. QPPs can be thought of as rhythmic waves that are a combination of standing and traveling waves. Originally described in rats in Majeed et al.,^17^ QPPs have since been detected in several human datasets.

**Fig. 2:**
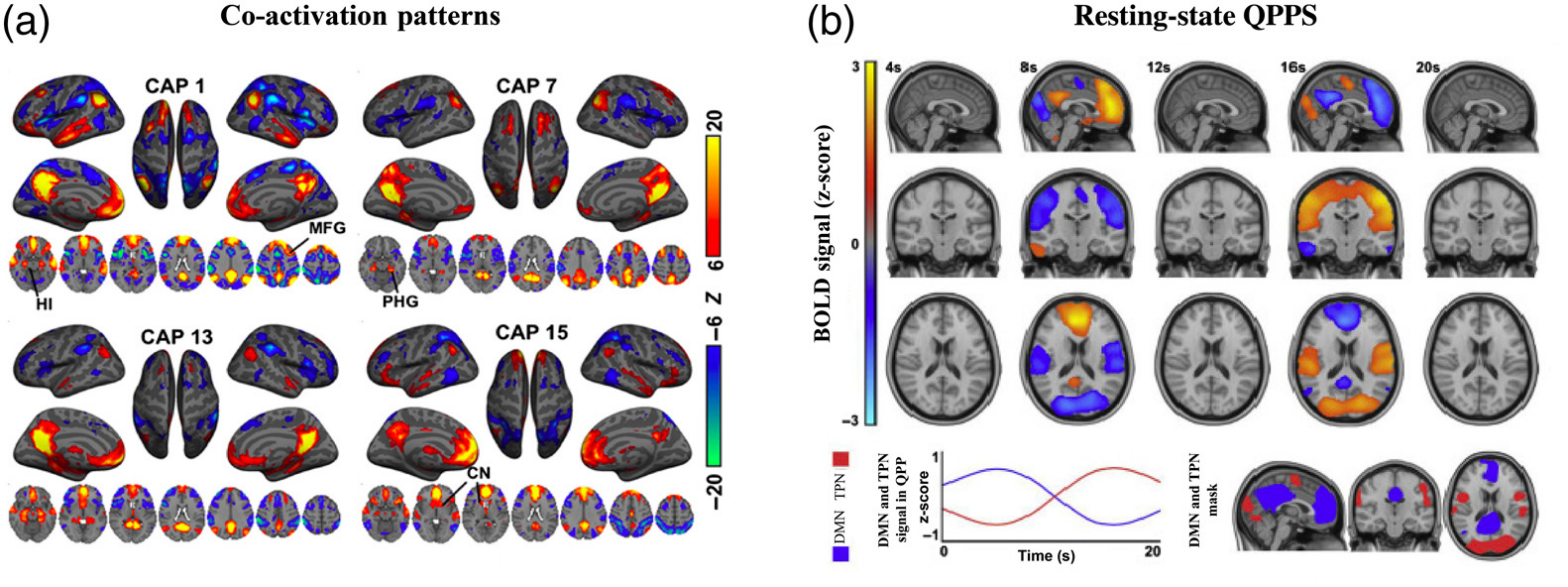
(a) CAPs showing distinct co-activation in “task-positive” areas and consistent deactivation in “task-negative” areas. Reprinted from Ref. 23. (b) Spatiotemporal patterns seen in the resting-state QPP. Reprinted with permission from Ref. 16.

A major feature of QPPs is that they show a reliable anti-correlation in propagating activity between the well-described default mode networks (DMNs) and task-positive networks (TPNs) [Fig 2(b)].^16^^,^^19^ The DMN and TPN are intrinsic connectivity networks that are traditionally associated with either introspective self-referential cognition (mind wandering) or with the conscious direction of attention or processing of incoming stimuli, respectively.^16^^,^^20^ QPPs also contribute to normal functional connectivity and the level of anti-correlation typically seen between the DMN and TPN.^16^ The contribution of QPPs to typical functional connectivity in attentional networks may shed light on the etiology of diseases and brain states related to disrupted attention. Work by Abbas et al.,^16^ for example, found a reduced contribution of QPPs to functional connectivity in individuals with attention-deficit/hyperactivity disorder.

The neural origins and mechanisms underlying the generation of QPPs is an area of active research, but early work with local field potentials and resting-state fMRI (rs-fMRI) in rodents suggests that QPP activity is correlated to infraslow electrical activity.^18^^,^^21^ Spontaneous infraslow patterns have also been detected in mice using wide-field voltage-sensitive dye imaging, but specific mechanisms underlying the generation of infraslow patterns are still unclear.^22^ A combination of multimodal and/or cell-type specific techniques in rodents may provide the best approach to better understanding the origins and role of QPPs in dynamics and functional connectivity.

#### 4.2.2 Co-activation patterns

Co-activation patterns, or CAPs, are brief periods, a few seconds long, of spontaneous co-activation or co-deactivation between different brain regions. These CAPs represent recurring instantaneous brain states near the temporal resolution of a single fMRI time point.^24^ CAPs analysis uses single fMRI volumes rather than time series as the basic units of analysis.^23^^,^^25^ The method use a k-means algorithm to cluster and average activities at different spatial and temporal points.^23^^,^^24^^,^^25^ CAPs often appear in pairs, in which one group of areas is activated and another is deactivated in one CAP and the inverse pattern occurs in another CAP. These complementary patterns might be considered different phases of a standing wave, similar to the standing waves that were observed with optical imaging.

The CAPs method was initially used in rs-fMRI data to decompose spontaneous rs fluctuations of well-characterized networks into distinct CAPs that happen on a smaller dynamic timescale.^23^ For example, the CAPs method was employed by Liu et al.^23^ to analyze dynamics within the DMN. In this study, it was found that the DMN contained multiple CAPs and diverging activities of the nuclei within the canonical DMN that are not detected with traditional static functional connectivity analysis. Some, but not all, of the CAPs detected show correlated activation that resembles traditional seed-based DMN activation maps or independent components analysis maps [Fig 2(a)]. However, some of the CAPs showed deactivation or coactivation only within specific subsets of the DMN, such as the hippocampus, parahippocampal gyrus, and caudate nucleus.

In addition to rs analyses, CAPs have also been used in task-based fMRI datasets to investigate functional networks. For example, using CAPs analysis, one study found that the typical DMN connectivity and anti-correlation with the TPN was robust across tasks and during rest.^49^

CAPs provide another innovative option for the dynamic analysis of functional datasets to reveal possible patterns and mechanisms that may be missed by traditional static connectivity analysis. However, as with QPPs, or with other types of more recently described dynamic patterns of functional connectivity, the exact neural basis of CAPs is still not well understood.^16^^,^^25^ Unlike optical imaging or electrophysiology, fMRI is sensitive to hemodynamics, which means systemic oscillations of the vasculature can influence the signal, along with physiological cycles such as respiration or cardiac pulsation, and this makes it more difficult to interpret the patterns that are observed. It is possible that more than one type of neural activity can contribute to the CAPs and other types of spontaneous signals seen in fMRI.^25^ However, the presence of propagating and standing waves of activity is consistent with prior findings in more specific modalities.

#### 4.2.3 Deep learning of spatiotemporal trajectories

Deep learning and artificial neural networks have recently been used to identify characteristic spatiotemporal trajectories in rs-fMRI data.^39^ In Zhang et al.,^39^ a variational autoencoder was used to identify a relatively small number of spatiotemporal features of rs-fMRI, which may act as the building blocks for spatiotemporal patterns such as QPPs and CAPs.^39^ Some of the spatiotemporal trajectories modeled with this method closely resemble QPPs and CAPs, but others may represent unique spatiotemporal patterns. This autoencoder method also found anti-correlation between the activity of the DMN and TPN, consistent with QPP and CAPs analysis methods. As with other MRI-based methods, the spatiotemporal trajectories exhibit aspects of standing and traveling waves.

Sobczak et al.^50^ also used a neural network approach to investigate fMRI dynamics. Specifically, recurrent neural networks were trained on vessel-specific rs-fMRI signals to predict the temporal evolution of rs-fMRI dynamics up to 10 s ahead. This approach used both principal components analysis and independent component analysis to reduce the dimensionality of the data and identify independent components in underlying brain networks. This was done on both rat data and human connectome project data.^50^ The studies demonstrate the potential for deep learning to provide additional insight into the spatiotemporal evolution of brain activity.

### 4.3 Spatiotemporal fMRI Pattern Summary

Dynamic fMRI analysis of QPPs, CAPs, and other spatiotemporal trajectories already shows great promise in detecting unique spatiotemporal patterns that are missed by traditional static fMRI analysis. Each of these approaches has led to new insights into spatiotemporal dynamics and has generated a validation of the findings shared between the approaches. For example, QPPs, CAPs, and the variational autoencoder approach to spatiotemporal patterns all validate some aspects of DMN/TPN anti-correlation. A common drawback of all of these spatiotemporal analysis methods is a lack of understanding of the origins of the patterns.

As previously mentioned, multimodal approaches in animals and humans provide the most promising route to a better understanding of the neural basis of these patterns and to a translational impact. Pairing fMRI with cell-type-specific imaging techniques such as optical imaging, opto/chemo-genetics, or electrophysiology in animal models will lead to better models of the neural substrates of cortex-wide spatiotemporal patterns and give experimental insight into circular causality across scales.

## 5 Electroencephalogram

### 5.1 Source of Signal

The primary source of the signal captured by electroencephalography (EEG) recordings is the electrical activity generated by postsynaptic potentials in the apical dendrites of cortical pyramidal neurons.^51^^,^^52^ Because the sensors are located relatively far from the neurons, the EEG signal represents the summed activity of a large number of neurons spread across the cortex.

EEG’s sensitivity to electrical events on the order of postsynaptic potentials means it has significantly higher temporal resolution than fMRI. EEG can capture electrical events ranging from millisecond to hundreds of milliseconds.^44^ Although the temporal resolution of EEG is very fast, the spatial resolution of EEG is one of its major drawbacks. EEG relies on the placement of scalp electrodes, with a higher number of electrodes resulting in better signal localization and resolution.^52^ Even with a high number of scalp electrodes (ranging from 4 to 256 electrodes in some studies^53^), the spatial resolution of EEG is limited to between 10 and 20 mm and is limited to electrical activity at the cortical surface, as the sources rely on superficial postsynaptic potentials.^44^^,^^51^

Individual postsynaptic potential events as measured with EEG are extremely small and do not result in a large signal.^51^ However, when a large number of excitatory or inhibitory postsynaptic potentials [excitatory post - synaptic potentials (EPSPs) or inhibitory post - synaptic potentials (IPSPs], respectively) occur with synchrony, the effects summate and can result in detectable EEG waves. The frequency and amplitude of such waves due to variation in underlying synchrony constitute the primary signal that can be used in EEG to measure differences across brain states or disease states.^51^ A number of important waveforms and patterns have been detected with EEG that correlate with different brain states, levels of consciousness, sensory inputs, or disease states. However, the canonical oscillatory bands of EEG such as delta, theta, alpha, or beta waves are not the focus of this review. Instead, EEG microstates will be discussed, as their spatiotemporal characteristics are similar to CAPs and comparable optical spatial patterns.

### 5.2 Dynamic Patterns

EEG microstates are spontaneous spatiotemporal patterns of electrical activity occurring on the sub-second timescale and lasting between 80 and 120 ms.^26^^,^^54^ These microstates are described as being quasi-stable for those brief sub-second periods. In other words, EEG microstates are a form of standing wave. EEG microstates are normally measured during rs (eyes closed). Somewhat similar to QPPs or CAPs observed in rs-fMRI, EEG microstates are global patterns that reliably recur temporally and topographically across subjects.^26^^,^^27^

Similar to functional networks such as the DMN or TPN, defined in fMRI studies, EEG microstates have also led to the description of canonical brain states. Four canonical classes of microstate maps that are reliable across studies have emerged over time.^54^^,^^55^^,^^28^ Changes in the typical spatiotemporal configuration of these activation maps have been observed during different cognitive and neurological states [Fig 1(c)]. For example, a 1998 study by Lehmann et al.^56^ presented human subjects with a tone at a random interval and then asked them to relay what was going through their mind just before they heard the tone. Thoughts were then classified as visual or abstract thinking and then compared with the EEG microstate topography at the moment preceding the report of visual or abstract thinking. The microstate associated with visual thoughts closely resembled the second canonical EEG microstate, which has been implicated in resting visual network activity.^54^^,^^56^ In a related study, EEG microstates were found to differ significantly when subjects were presented with words associated with either imagery or abstract meaning.^55^ Studies such as these have led to the suggestion that microstates may be the fundamental building blocks or units of cognition or mentation.^54^ In addition to normal cognition or mentation, EEG microstates have also been shown to differ in psychiatric disease states such as schizophrenia [also see Fig 1(c)].^28^

As with fMRI, combining multiple modalities in both humans and animals may be the most promising route to an integrated understanding of the origins of spatiotemporal patterns such as microstates detected in EEG. In humans, studies have paired EEG with simultaneous fMRI to compare EEG microstates with BOLD activation maps, giving some insight into the source localization of EEG microstates.^29^^,^^30^ To better understand the neural origins of EEG microstates, EEG could also potentially be paired with fMRI or other more invasive and cell-type-specific approaches in transgenic animal models. Multimodal animal approaches could shed light on the cross-species persistence of the four canonical EEG microstates seen in human studies and provide experimental tools to investigate their origins.

## 6 Possible Sources of Patterns

Spontaneous rs patterns travel along brain networks at multiple scales modulating neural activity. These spatiotemporal patterns transverse the mammalian neocortex through its massively interconnected synaptic network in which a vast majority of excitatory synapses onto cortical excitatory neurons come from other cortical excitatory neurons.^57^^,^^58^ As a consequence cortical and thalamic neurons can initiate and sustain patterned network activity. In some instances, this network activity results in periods of intense synaptic activity, termed “up” states, and almost complete silence, termed down states. It is the relationship between patterned network activity and anatomical architecture that allows for brain operations to be carried out simultaneously at multiple temporal and spatial scales.^59^

On a mesoscopic network scale, the thought behind the initiation of cortex-wide activity is split into two theories: initiation from persistently active pacemaker cortical neurons or stochastic initiation by temporal summation of spontaneous synaptic activity.^60^ In both instances, however, pyramidal cells in cortical layer 5 are known to be acting as key players in driving synchronous network activation due to their dense recurrent synaptic connectivity. In the first *in vitro* recordings of slow oscillations, it was shown that multiunit activity was the strongest and earliest in layer 5.^6^ When synaptic connections were severed via a horizontal cut through layer 4, the lower cortical layers still generated the slow oscillation, whereas the upper layers generated this activity infrequently or not at all. The importance of layer 5 cells in initiating up states was demonstrated later *in vivo* work and through optogenetic manipulation of layer 5 cells *in vivo*.^7^^,^^61^ Many layer 5 pyramidal cells exhibit intrinsic rhythmic activity that resonates at frequencies <15  Hz following the short depolarizing or hyperpolarizing current pulses, which could facilitate the slow oscillation seen in the entire cortical network. However, it is important to note that other sources have been found to contribute to slow oscillation activity. For example, work in humans and primates has shown that “fidgeting” type movements result in slow-wave signals.^62^ Small movements of the face and limbs such as slight head movement, blinking, swallowing, etc., can occur in the absence of any stimulation and contribute to slow spatiotemporal fluctuations in functional activation.^62^

For a macroscopic network scale, neural recordings such as those obtained from EEG, wide-field optical imaging, or fMRI capture the effects of interaction among billions of neurons across the cortex. Connections across large-scale populations are mediated by cortical white matter fibers, which connect nearby regions or long-range areas.^1^ There are distance-dependent delays as signals have to transverse axonal conduction up to tens of milliseconds.^63^ These delays influence the emergence of spatiotemporal patterns at the macroscopic level, including radial and spiral waves that can be found in networks of coupled oscillators.^1^^,^^15^ Thus the macroscopic structural network of the brain can contribute to the coordination of mesoscale patterns into whole-brain patterns. Moreover, many neuromodulatory nuclei (e.g., locus coeruleus, raphe nuclei) have widespread, spatially distributed inputs to the rest of the brain, which may further organize the mesoscale activity into macroscale trajectories.^64^

## 7 Future Directions

### 7.1 Complementary Multimodality Studies

Each of the aforementioned modalities has its own strengths and weaknesses as outlined in Table 1. To leverage their individual strengths, multimodal imaging approaches have become increasingly popular. Multimodal imaging has the promise of allowing for not only the study of how different signals are related across scales but also a better understanding of how the observed dynamic patterns are influenced across these scales. There is also no known timing for wide-scale intrinsic brain activity, and simultaneous measurement at different spatial and temporal scales is needed to bridge this gap.

EEG is a valuable multimodal tool for correlating electrophysiological events with dynamic patterns. Recent efforts to better understand the origins of EEG microstates have paired EEG with fMRI, in which source localization of microstates can be inferred by comparing simultaneously measured BOLD activation.^29^^,^^30^ Simultaneous recordings of EEG microstates and BOLD fMRI show that some canonical rs networks from fMRI such as the DMN correspond to microstate associated networks as measured with EEG.^29^^,^^30^ Beyond microstates, other multimodal studies have compared the infraslow dynamics of multichannel EEG with BOLD fMRI. For example, using a multimodal approach with simultaneous EEG and fMRI in human subjects, it is possible to correlate the infraslow dynamics of scalp potentials (0.01 to 0.1 Hz) with the infraslow dynamics of the BOLD signal. Using this approach, it has been shown that scalp potentials from EEG correlated strongly to fMRI/BOLD dynamics temporally and spatially.^65^^,^^66^ Anatomically, EEG dynamics correlate with the BOLD activity of well-defined intrinsic connectivity networks in fMRI including the DMN and the TPN.^65^ Infraslow dynamics have also been studied using simultaneous LFP and fMRI.^42^ A spontaneous BOLD signal at the recording sites was found to exhibit significant localized correlation with the infraslow LFP signals as well as with the slow power modulations of higher-frequency LFPs (1 to 100 Hz) with a delay comparable to the hemodynamic response time.^42^

Simultaneous multimodal methods to measure complementary features of activity using optical imaging and fMRI have also been described.^81^^–^^70^^,^^71^ Wide-field optical imaging provides the advantage of covering a large field of view while measuring cell-type-specific signals.^67^^,^^69^^,^^70^ There have been great advances made in understanding the functional roles of different cell types, but what remains unknown is how the activity at the cellular scale affects the activity measured at a global scale recorded with fMRI. Because the brain exhibits structured activity at all scales, a better understanding of how different levels contribute to the system as a whole is still needed. The combination of wide-field optical imaging with fMRI to study how different cell types influence hemodynamic regulation is particularly promising.^67^^,^^70^^,^^72^ Another potential benefit of simultaneous optical imaging and fMRI is that the whole brain coverage of fMRI provides some information about activity in subcortical areas, which cannot be observed optically but which plays an important role in the generation of coordinated brain activity.

One of the main limitations of wide-field optical imaging is that it does not allow for the resolution of single neurons, capillaries, or subcortical nuclei.^69^ Fiber photometry is another promising approach that has been increasingly used for multimodal fMRI studies.^72^^–^^76^ Fiber photometry allows for cell-type-specific fluorescence imaging such as wide-field optical imaging with the placement of one or more fibers that can target specific neural populations or regions of interest.

Multimodal fiber photometry has been successfully paired with fMRI in rats, as in Liang et al.^75^ and Tong et al.,^73^ and in mice, as in Wang et al.^72^ and Schlegel et al.^76^ The targeting specificity and subcortical reach of fiber photometry mean that it can be used to compare multimodal signals obtained in different nuclei of interest. This can be done in parallel animals or with multiple fibers within one animal.^77^ Fiber photometry tracts can be multichannel and capture signals from multiple fluorophores or physiological sources at once.^74^ Chao et al.^74^ recently used a fiber photometry/fMRI approach to simultaneously measure GCaMP6f fluorescence, photometry-based cerebral blood volume (CBV), and fMRI CBV. This fiber photometry approach allowed them to estimate hemodynamic response functions in different subcortical nuclei, an analysis that could not be made with a superficial approach such as wide-field optical imaging.

A multimodal fiber-optics approach was used in Schwalm et al.^78^ to study slow-wave brain dynamics. Using fiber photometry to record calcium indicators in populations of the cortex and thalamus in anesthetized rats with simultaneous BOLD fMRI, they found that the calcium activity of the entire cortex was related to the generation of slow-wave BOLD activity.^78^ In another fiber optics example, Pais-Roldán et al.^79^ used fiber photometry Ca+2 imaging and BOLD fMRI to relate pupil dynamics to the global fMRI signal and found a positive correlation between pupil dilation and the activity of a population of noradrenergic cells in the ventral brainstem. He et al.^80^ used fiber optics with simultaneous fMRI to investigate the relationship between BOLD, CBV, and neuronal activity and found that the Ca+2 signal from neurons is linked to intrinsic cerebral vascular oscillations.

Given that complex brain-wide spatiotemporal patterns are unlikely to be driven by the activity of a single brain’s nuclei, the advantage of targeting multiple subcortical nuclei makes multimodal fiber photometry fMRI studies an excellent multimodal approach to studying brain dynamics. (For a more in-depth review of the multi-modal application of fiber optics and optogenetics in fMRI studies, see Albers et al.^81^)

An alternative approach that has been used to track Ca+2 activity in relation to the BOLD fMRI signal is the use of magnesium-enhanced MRI (MEMRI) ion imaging.^82^ For example, Duong et al.^82^ used anesthetized rats and MEMRImagnesium Ca+2 imaging to show that synapse dependent Ca+2 signal was correlated with BOLD and cerebral blood flow. The changes detected with MEMRI are much slower than those observed with methods such as optical imaging, often taking 24 to 48 h to show the enhancement throughout a pathway.^83^ The slower time scale makes it better suited to imaging long-term changes rather than the fast intrinsic dynamics that are the primary focus of this review.

### 7.2 New Imaging Tools

Ideally, to understand the relationship of neural activity across spatial and temporal scales, recordings should be made simultaneously from all neurons in the brain. To date, no imaging modality is capable of this task. Some, such as fMRI, obtain whole-brain coverage but lack sensitivity to individual neurons; others, such as electrophysiology, can detect activity in a single neuron but have limited spatial coverage. This gap is well-recognized in the field of neuroimaging, and improved methods that more closely approach the ideal case are constantly being developed, in part due to support from the BRAIN initiative. Larger microelectrode arrays, optical reporters that provide greater depth sensitivity, and higher resolution fMRI studies have all had a positive impact on the coverage and/or resolution that can be obtained.

Functional ultrasound (fUS) also presents a unique and promising approach to studying large-scale spatiotemporal patterns. Initially developed in rats,^84^ and relying on a thinned-skull cranial window, Osmanski et al.^84^ found that fUS is capable of increased spatial and temporal resolution compared with fMRI, with spatial resolution on the order of 100  μm and a temporal resolution of 2 ms. The equipment for fUS is also significantly more portable and less expensive than fMRI. Recently, fUS has been successfully used in mice to measure the activity of the default mode network.^85^ Ferrier et al.^85^ found that, under an awake or slightly sedated state, mice showed deactivation of hubs of the DMN, including the retrosplenial cortex, validating the use of fUS to study functional networks and spatiotemporal dynamics in mouse models. fUS has also been used in human neonates.^86^ Baranger et al.^86^ showed that fUS can be used as an effective and portable bedside method for measuring functional connectivity in human neonates in both healthy and diseased states. fUS has also been combined with EEG in human neonates to study sleep phases and epilepsy.^87^ In both animal models and human neonates, fUS provides a portable and cost-effective alternative to fMRI that offers improved spatial and temporal resolution. However, fUS is limited in its potential to capture whole-brain activity in adult humans because of its poor penetration through the skull, and it is better suited to rodents with a cranial window or human neonates before the adult formation of the skull is complete.

### 7.3 Role of Spontaneous Activity in Brain Function

Many experimental approaches to understanding functional aspects of different stimuli or tasks rely on averaging large numbers of trials to effectively suppress background activity and highlight only activity that is repeatable across trials. However, there is growing appreciation that the dynamics of ongoing, systems-level activity influence activity in different trials and account for much of the variability that is observed. As an example, infraslow dynamic patterns detected with EEG have been shown to have predictive power for the response to incoming somatosensory input. In a 2008 study by Monto et al.,^88^ human subjects received a weak electrical stimulus to the right index finger and their ability to detect the stimulus was correlated with the phase and amplitude of infraslow EEG dynamics. It was found that the likelihood of a subject perceiving a weak stimulus was significantly higher during the rising phase of the infraslow EEG fluctuations compared with the other phases.^52^ Studies such as this demonstrate the influence of spontaneous dynamics on perception and task performance and make it clear that the “circular causality” between dynamics at different scales has important implications for human behavior.

More recently, Iemi et al.^89^ also used EEG to study the variation in response to stimuli based on internal states. Specifically, Iemi et al.^89^ measured the variation in the event-related potential (ERP) compared with the EEG waveform present in the subject when the stimulus was received. They found that the ERP changed if the stimulus (black and white checkerboard images) was applied during strong alpha or beta waves.^89^ In rodent studies of the role of spontaneous activity in modulating stimulus-response, Karvat et al.^90^ used a closed-loop LFP system to test the hypothesis that beta-band activity can mask the response to an externally applied stimulus. Using LFP, beta-band activity was measured and compared with sensory-evoked responses in rats. It was found that beta-power activity in the rat somatosensory cortex was anti-correlated with sensory-evoked responses. In other words, the incoming sensory input could be masked depending on the band of spontaneous background activity occurring in the somatosensory cortex.

Studies such as these provide further evidence that spontaneous background activity heavily modulates or even masks perception and reaction to external stimuli. Further work is needed across modalities and models to better characterize the interplay between multiscale, ongoing activity and behavioral outcomes such as perception or task performance.

### 7.4 Astrocytic Contribution to Brain Wide Patterns

Recently, GECIs have been combined with fiber photometry and fMRI in mouse models to explore the contribution of astrocytic activity to the BOLD signal and brain-wide patterns. For example, Gray et al.^77^ used simultaneous fiber photometry in the cerebellum and V1 of anesthetized mice to show that astrocytic calcium signaling tracks the dynamics of noradrenergic neurons in response to stimuli and that astrocytes may act as amplifiers of vigilance states. In the thalamus, Wang et al.^72^ used simultaneous GECI optical fiber recording and fMRI in anesthetized rats to relate astrocytic calcium signal to the BOLD signal both at rest and in response to a stimulus. They found that the evoked astrocytic Ca+2 signal positively correlated with the BOLD signal, whereas the rs or intrinsic Ca+2 signal negatively correlated with the BOLD signal.^72^

Going forward, multimodal fMRI approaches are the most promising route to a better understanding of the role of astrocytes in brain-wide spatiotemporal dynamics. In addition to pairing cell-type-specific fiber photometry and optical imaging with fMRI, chemogenetics and optogenetics provide experimental options to drive the activity of astrocytic Ca+2 signals and study their role in brain-wide dynamics.

### 7.5 Complex Systems Analysis

With coordinated patterns of activity at multiple spatial and temporal scales, the brain has all of the hallmarks of a complex system and shares features with other complex systems (e.g., global climate). New methods for characterizing these complex systems and their cross-scale interactions are continually being developed, and each new tool provides another view of the functional organization of the brain. For example, some research suggests that the brain as a complex system self organizes into a critical state. In this state interactions between system components lead to scale-invariant events that are beneficial for the system performance by obtaining an optimal balance of stability and flexibility.^91^^,^^92^ These tools and concepts can inform further research that aims to drive an individual brain into particular states using invasive or noninvasive brain stimulation technology, moving the field closer to truly personalized medicine.

## 8 Conclusion

Through the use of various recording and imaging techniques, considerable progress has been made in elucidating the cellular and network mechanisms involved in coordinated brain-wide dynamics. This review has outlined four commonly used imaging modalities that have provided unique insights at various scales, allowing for a better understanding of the self-organization of the brain.

The emergence of self-organization has two directions: the upward or local to global causation and the downward or global to local causation. This results in the circular causality of spontaneous brain activity. The spatiotemporal patterns of this spontaneous brain activity are hard to tackle, in part, because spontaneous activity acts in the absence of outside influences. The patterns found suggest that the largest amplitude and most regular spontaneous oscillations occur at the seemingly “wrong” time, during sleep or when the brain is otherwise disengaged from the environment.^93^

There are still many further avenues for investigation of the mechanisms and functions of these spatiotemporal dynamics. Promising future approaches can provide increasingly informative experimental access to dynamic states at different resolutions, providing a path forward for elucidating the patterns involved in creating dynamical brain states that are important in health and disease.
